# Cataract Surgery in Anterior Megalophthalmos: A Review

**Published:** 2015

**Authors:** Virgilio GALVIS, Alejandro TELLO, Carlos M. RANGEL

**Affiliations:** 1 Centro Oftalmológico Virgilio Galvis, Floridablanca, Colombia; 2 Fundación Oftalmológica de Santander FOSCAL, Floridablanca, Colombia; 3 Universidad Autónoma de Bucaramanga (UNAB), Bucaramanga, Colombia; 4 Universidad Industrial de Santander, Bucaramanga, Colombia

**Keywords:** Iris-claw intraocular lens, megalocornea, megalophthalmos

## Abstract

Anterior megalophthalmos is characterized by megalocornea associated with a very broad anterior chamber and ciliary ring elongation. It is also called X-linked megalocornea. It is accompanied by early development of cataracts, zonular anomalies, and, rarely, vitreoretinal disorders. Subluxation of a cataract can occur in cataract surgery because of zonular weakness. In addition, in most patients, standard intraocular lens (IOL) decentration is a risk because of the enlarged sulcus and capsular bag. These unique circumstances make cataract surgery challenging. To date, several approaches have been developed. Implantation of a retropupillary iris-claw aphakic intraocular lens may be a good option because it is easier than suturing the IOL and can have better and more stable anatomic and visual outcomes, compared to other techniques.

## INTRODUCTION

**Figure 1:**
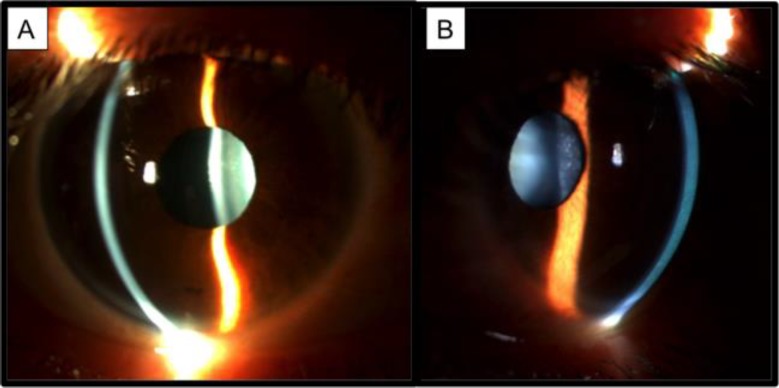
Anterior megalophthalmos presents with megalocornea and a very deep anterior chamber. Early onset cataracts are a common finding (Photos are kindly provided by Dr. Virgilio Galvis).

In 1914, Seefelder initially described anterior megalophthalmos, as cited by Wright (1). It is characterized by megalocornea, which is associated with a very deep anterior chamber and ciliary ring elongation (Figure 1) (1). The pathogenesis of anterior megalophthalmos remains unknown. It results from keratodysgenesis and iridogoniodysgenesis, or both (2). X-linked recessive inheritance exists in 50% of patients, autosomal transmission in 40% of patients, and it is sporadic in the remaining 10% of patients. Men constitute approximately 90% of patients. Gene linkage data have suggested that the X-linked megalocornea locus maps in the region Xq12-q26 (3). Other diseases associated with anterior megalophthalmos are Marfan’s syndrome, trisomy 21, Apert syndrome, mucolipidosis type 2, and Walker Warburg syndrome (4,5).

Early appearance of cataracts and subluxation of the crystalline lens, are the leading causes of decreased vision in these patients. Cataracts usually develop between the ages of 30 and 50 years (3,6). Besides cataracts and zonular anomalies, these patients also present with other anterior segment abnormalities: atrophy of the iris, hypoplasia of the pupil dilator muscle, transillumination defects of the iris, pigmentary dispersion, mosaic corneal dystrophy, anterior embryotoxon, myopia, iridodonesis, miosis, inadequate pupillary dilatation, retroposition of the lens-iris diaphragm, shortening of the vitreous cavity, a very wide angle on gonioscopy with band broadening of the ciliary body, and excessive mesenchymal tissue in the anterior chamber angle (7). Posterior segment abnormalities in the eye also have been described: vitreous fibrillar degeneration with liquefaction, optically empty vitreous with strands, peripheral retinal degenerations (lattice), spontaneous vitreous hemorrhage, peripheral retinal neovascularization, retinal breaks, and retinal detachment (8, 9). In patients with corneal enlargement, differential diagnoses include megalocornea, megalophthalmos, congenital glaucoma, and keratoglobus (1-29) (Table 1). The principal characteristics of megalocornea are bilateral, non-progressive enlargement of the cornea, which maintains transparency; a hereditary disorder, mostly with an X-linked recessive inheritance; and lack of any evidence of glaucoma (10,11).

Anterior megalophthalmos is very similar to megalocornea, but these eyes also show enlargement of the lens–iris diaphragm and ciliary ring, in addition to corneal abnormalities (1-4). The differentiation between isolated megalocornea and anterior megalophthalmos is not a straightforward task, and many authors have reported on megalocornea cases that were actually anterior megalophthalmos cases (17, 20, 22, 25, 26, 31, 35).

Megalocornea and anterior megalophthalmos can be differentiated from congenital glaucoma by the transparency of the cornea. Both conditions have a normal endothelial cell count and morphology (13, 28), whereas congenital glaucoma presents with polymegethism, pleomorphism, and decreased cell density (30). In anterior megalophthalmos, the intraocular pressure and optic nerve are normal. Congenital glaucoma is usually progressive and asymmetric and symptoms such as photophobia and tearing frequently occur, along with the characteristic sign of single or multiple horizontal or radial ruptures in Descemet’s membrane (i.e., Haab’s striae) (10). In congenital or infantile glaucoma, the axial length is elongated mostly because of the expansion of the posterior segment; in megalocornea and megalophthalmos, the axial length is normal and only the anterior segment of the eye is enlarged (10, 12).

Another condition that must be distinguished from anterior megalophthalmos is keratoglobus. Keratoglobus is a corneal ectasis that, like megalocornea and megalophthalmos, causes bilateral bulging globoid corneas. However, unlike megalocornea and megalophthalmos in which the corneas have a normal curvature (14-29) and normal or mildly decreased thickness (15, 16, 20, 22, 23), the corneas in keratoglobus are remarkably thin and the enlargement of the corneal diameter is small (10). Only two cases of anterior megalophthalmos have been described with significant corneal thinning (22, 24).

**Table 1:** Differential Diagnosis of Megalocornea (10)

**FEATURE**	**CONGENITAL GLAUCOMA**	**MEGALOCORNEA/MEGALOPHTHALMOS**	**KERATOGLOBUS**
**Onset**	Congenital or Infantile	Congenital	Congenital
**Inheritance **	Recessive	Usually X-linked	Recessive
**Bilaterality**	Assymetrical	Possible assymetrical	Bilateral
**Corneal Thickness**	Variable	Moderately thin	Very thin
**Corneal Diameter**	Progressive enlargement	Large and stable (> 13 mm)	Normal or slightly enlarged
**Anterior Chamber**	Deep	Very deep	Deep or very deep
**Iris Transillumination**	Absent	Frequently present	Absent
**Posterior Iris Bowing**	Minimal	Frequent and pronounced	Absent
**Pigment Dispersion**	Absent	Frequent	Absent
**Corneal Curvature**	Around normal	Around normal	Very steep
**Refraction**	Myopia variable	Variable, usually low refractive errors, but may range from high hyperopia to high myopia.	High myopia
**Gonioscopy**	Abnormal	Band broadening of the ciliary body and excessive mesenchymal tissue	Iris processes
**Sistemic Associations**	Sturge-Weber, other pediatric syndromes	Marfan, Alport, Down, Mucolipidosis tipo II, pontocerebellar hypoplasia, Walker Warburg syndrome (rarely)	Ehlers-Danlos, Rubinstein-Taybi syndrome

## CATARACT SURGERY IN ANTERIOR MEGALOPHTHALMOS

Some challenges of cataract surgery for patients with anterior megalophthalmos are the extremely deep anterior chamber, which may make visualization difficult and surgical maneuvers more challenging; all landmarks and dimensions are abnormal, which can make estimating the capsulorhexis size difficult; and zonular anomalies and a large capsular bag can lead to complications such as posterior capsule rupture and vitreous loss. In addition, intraocular lens (IOL) decentration related to the oversized capsular bag is common (14, 15, 19, 20, 25, 26, 29, 31-33). In reviewing the literature from 1984 to date, we found 30 patients (representing 51 eyes) who had anterior megalophthalmos and underwent cataract surgery (Table 2). We chose not to include reports of cases before that year because of the important differences in surgical techniques, compared to "modern” cataract surgery. Different approaches have been used to avoid IOL instability such as leaving patients aphakic; using contact lenses or aphakic glasses for visual rehabilitation (29); secondary sutured IOL implantation (29); large custom-made IOLs, which are an excellent alternative but are not commercially available, can be difficult to obtain, and are expensive (23); phacoemulsification with anterior optic capture of a three-piece IOL (34); and IOL suturing techniques (15, 16, 25, 26, 29, 32, 35). Iris-sutured IOLs may become loose in eyes with anterior megalophthalmos (25).

**Table 2:** Reported Cases of Cataract Surgery and IOL Implantation in eyes with Anterior Megalophthalmos (1984–2014)

**Author, Year**	**Case/Eye**	**Surgical Technique/IOL**
**Neumann, 1984**	Case 1:OU (Neumann, 1984)	First eye: ECCE + IOL in sulcus: decentration. It was removed and a Medallion IOL sutured to iris. In the fellow eye: Medallion IOL sutured to iris.
	Case 2 :One eye (Neumann, 1984)	One eye: ECCE + Medallion IOL sutured to iris
**Kwitko, 1991**	Case 1:OU (Kwitko, 1991)	OD: ECCE + IOL (14 mm ) in sulcus. 6 ms POP: mild inferior decentration. 1 yr POP: Retinal detachment. OS: ECCE + IOL (14 mm) in sulcus. 1 yr POP: mild superior decentration. 18 ms POP: Retinal detachment. Following retinopexy, IOL subluxation.
	Case 2: OD (Kwitko, 1991)	OD: ECCE + IOL (18 mm). Good evolution.
**Dua, 1999**	Case 1: OU (Dua, 1999)	OU: ECCE + IOL sutured to iris and anterior capsule.
**Javadi, 2000**	Case 1: OU (Javadi, 2000)	OU: ECCE + standard PMMA IOL in the bag (13.5 mm length, 7.0 mm optic)
	Case 2: OU (Javadi, 2000)	OD: ECCE + standard PMMA IOL (13.5 mm length, 7.0 mm optic) in the bag (can-opener capsulotomy). Decentration. OS: ECCE+ standard PMMA IOL in the bag (13.5 mm length, 7.0 mm optic)
	Case 3: OD (Javadi, 2000)	OD: ECC + LIO. Zonular dialysis, anterior vitrectomy and AC IOL. Significant pseudophacodonesis. Retinal detachment 3 ms POP.
	Case 4: OS (Javadi, 2000)	OS: Phacoemulsification+ standard PMMA IOL (13.5 mm length, 7.0 mm optic) in the bag.
**De Sanctis, 2004**	Case 1: OU (De Sanctis, 2004)	OD: Phacoemulsification+ foldable IOL (13.0 mm length) + capsular tension ring. Zonular dialysis. Mild superior decentration. OS: Phacoemulsification+ foldable IOL (13.0 mm length)
**Sharan, 2005**	Case 1: OU (Sharan, 2005)	OD: ECCE + aphakia OS: ECCE + aphakia. 10 yrs later secondary implantation: sutured AC IOL
	Case 2: OU (Sharan, 2005)	OD: ECCE + aphakia OS: ECCE + aphakia. 1 yr later secondary implantation standard IOL: decentration. Explantation and iris sutured IOL.
	Case 3: OU (Sharan, 2005)	OS: ECCE + Aphakia. Secondary implantation of custom made PMMA IOL (14 mm length). OD: ECCE + standard PMMA IOL (14 mm length).
**Basti, 2005**	Case 1: OD (Basti, 2005)	OD: sutured AC IOL. Decentration, instability. Explantation, and implantantion of a posterior chamber IOL sutured to iris
**Tsai, 2005**	Case 1: OD (Tsai, 2005)	OD: Phacoemulsification+ standard PMMA IOL (13.0 mm length, 6.0 mm optic) in the bag
**Oetting, 2006**	Case 1:OU (Oetting, 2006)	OU: Intracapsular extraction, aphakia. Late secondary implantation (20 yrs POP): iris-claw IOLs in AC. Refixation was required in OD
**Lee, 2006**	Case 1: OU (Lee, 2006)	OS: Pigmentary glaucoma. Previous trabeculectomy. Phacoemulsification + retropupillary iris-claw IOL OD: Phacoemulsification + retropupillary iris-claw IOL
**Vaz, 2007**	Case 1: OU (Vaz, 2007)	OU: Phacoemulsification+ custom made IOL (16 mm) in the bag
**Berry-Brincat, 2008**	Case 1:OU (Berry-Brincat, 2008)	OU: Phacoemulsification+ 3-piece foldable IOL in the bag. Decentration
**Assia, 2009**	Case 1: OU (Assia, 2009)	OU: Phacoemulsification + 3-piece standard foldable IOL in the bag. OD: scleral wound leak requiring resuturing.
**Welder, 2010**	Case 1:OU (Welder, 2010)	OU: Iris sutured IOLs. OS: Late instability, explantation and iris-claw IOL in AC.
**Zare, 2011**	Case 1: OS (Zare, 2011)	OS: Phacoemulsification+ standard three-piece acrylic foldable IOL in the bag
**Rekas, 2011**	Case 1: OU (Rekas, 2011)	OU: Phacoemulsification+ foldable IOL sutured to a capsular tension ring
**Galvis, 2012**	Case 1: OU (Galvis, 2012)	OD:Phacoemulsification+retropupillary iris-claw IOL OS:Phacoemulsification+ retropupillary iris-claw IOL *
**Hegde, 2012**	Case 1: OS (Hegde, 2012)	OS: Phacoemulsification+ standard PMMA IOL (13.5 mm length, 6.5 mm optic) in the bag
**Li , 2012**	Case 1: OD (Li, 2012)	OD: ECCE + standard IOL in the bag (can-opener capsulotomy). Decentration. Then, haptic suture of the IOL to posterior surface of the iris, anterior capsule and sclera
**Wang, 2012**	Case 1: OU (Wang, 2012)	OD: ECCE + standard PMMA IOL (13.5 mm length) in the bag. OS: Phacoemulsification+ CTR + standard foldable acrylic four square haptics IOL (10.7 mm length) in the bag. Decentration. Remove IOL, implantation AC iris-claw IOL
	Case 2: OD (Wang, 2012)	OD: ECCE + standard PMMA IOL (13.5 mm length) in the bag. Mild decentration. OS: ECCE + standard PMMA IOL (13.5 mm length) in the bag. Decentration.
	Case 3: OU (Wang, 2012)	OD: EECC + standard PMMA IOL (13.5 mm length) in the bag. Decentration. IOL explantation and implantation AC iris-claw IOL OS: EECC? + standard PMMA IOL (13.5 mm length) in the bag. Decentration.
**Jain, 2014**	Case 1: OU (Jain, 2014)	OU: Phacoemulsification (scleral tunnel) + anterior capsule capture 3-piece IOL

* The patient recently underwent surgery in his left eye at our institution. This eye is not included in the original case report.

Aphakic iris-claw lenses such as the Artisan lens (Ophtec, Groningen, the Netherlands) or Verisyse lens (Abbott Medical Optics Inc., Santa Ana, CA, United States) have also been used in patients with anterior megalophthalmos by implanting the lens in the anterior chamber (17, 26, 33) or fixating them in the posterior surface of the iris (27, 28).

**Figure 2:**
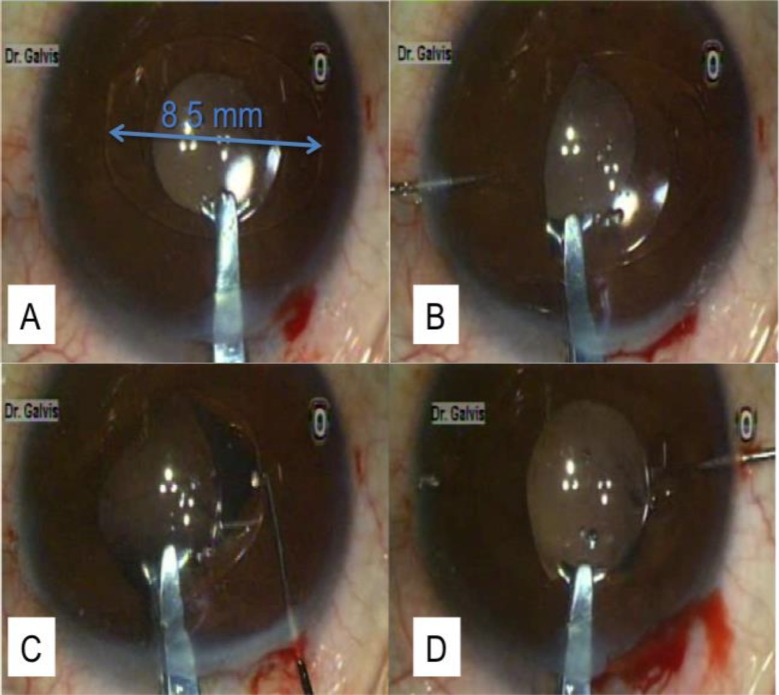
Aphakic Intraocular Lens Fixation with the Iris-claw Lens (Artisan lens; Ophtec, Groningen, the Netherlands)

In some patients with anterior megalophthalmos, a standard rigid polymethyl methacrylate (PMMA) IOL (total length, 13–13.5 mm) has been successfully used (19, 21, 33, 36). Standard multipiece foldable IOLs (18, 22) and single-piece foldable IOLs (20) have also been used. This suggests that enlargement of the capsular bag is not significant in all patients. Zare et al. (18) suggest using preoperative ultrasound biomicroscopy in anterior megalophthalmos to measure the actual size of the capsular bag to help decide whether a standard IOL is suitable. They were able to implant a standard three-piece foldable IOL in a patient with anterior megalophthalmos because ultrasound biomicroscopy revealed that the capsular bag diameter was normal, despite ciliary ring enlargement. However, most patients will present with a significantly enlarged capsular bag, which will cause a standard IOL to be at a high-risk of decentration. Iris-claw IOLs, which are fixated to the anterior stroma of the iris, are a good option in these patients. Fixation of an iris-claw IOL does not depend on the sulcus or on the bag; therefore, it is very useful in these patients when a large capsular bag may be problematic. However, in eyes with severe atrophy of the iris stroma, which was reported by Sharan et al. (29), fixation may be very difficult. Another technique using this type of aphakic IOL (i.e., Artisan lens or Verisyse lens) is fixating it to the posterior surface of the iris, as described in 1994 by Rijneveld et al. (37). This technique only became popular approximately one decade later, after Mohr described it again in 2002 (38). An advantage is that the optics of the IOL is much farther from the endothelium, and the anterior segment architecture is respected. Oetting and Newsom (17) implanted aphakic iris-claw lenses in the anterior chambers of two eyes in late secondary procedures. Wang implanted an iris-claw IOL in the anterior chamber as a secondary procedure in two eyes with a decentered previously implanted IOL: one eye had a foldable IOL and the other eye had a PMMA IOL (33). Welder and Oetting implanted an iris-claw IOL in the anterior chamber as a secondary procedure in a patient with a decentered iris-sutured posterior chamber IOL (26). Lee et al. (27) fixed the lenses retropupillary in two eyes with good results. We also implanted the iris-claw IOL in the posterior surface of the iris in both eyes of one patient (28). Like Lee et al. (27), we employed the posterior chamber fixation technique; however, unlike Lee, we performed the procedure using topical anesthetic eye drops instead of general anesthesia. Other differences from Lee is that we made a superior incision rather than a temporal incision and we used a spatula rather than enclavation needles for IOL fixation in the posterior surface of the iris through paracentesis incisions formed at the 3 o’clock and 9 o’clock positions (Fig. 2).

We believe that this type of lens, as suggested by other authors (17, 26, 27, 33) is an excellent alternative for patients with cataracts and anterior megalophthalmos because it eliminates the difficulties associated with instability of a lens in the bag or in the anterior chamber; and difficulties related to suturing it to the iris, anterior capsule or sclera, or performing techniques that are more demanding and have the risk of long-term instability. In addition, the retropupillary fixation of the aphakic iris-claw IOL may have the advantage of decreasing the risk of long-term endothelial cell loss.

(A) The intraocular lens (IOL) is already flipped with its anterior surface facing back toward the iris. The IOL length and the large limbus diameter are different. (B) The first haptic is slipped through the pupil, under the iris, and tilted so that the claw haptic raises the iris. A small amount of tissue is pressed back on the claw using a spatula to introduce it through the gap of the haptic. (C) and (D) The same procedure is performed with the second haptic. (Photos are kindly provided by Dr. Virgilio Galvis).

## CONCLUSION

Anterior megalophthalmos is characterized by megalocornea associated with a very deep anterior chamber and ciliary ring elongation. Cataract surgery is challenging because of the abnormalities in the anterior segment, especially the enlargement of the capsular bag and abnormalities of the zonules. Several approaches have been developed to date. Measuring the capsular bag diameter with ultrasound biomicroscopy may be useful to determine if the capsular bag is enlarged. In this situation, implantation of a retropupillary aphakic iris-claw IOL can yield excellent anatomic and visual outcomes.
